# 323. Comparative Effectiveness of Broad vs. Narrow Spectrum Antibiotics for Community-Acquired Pneumonia in Hospitalized Children with Medical Complexity

**DOI:** 10.1093/ofid/ofaf695.112

**Published:** 2026-01-11

**Authors:** Maheswari Ekambaram, Yun Li, Jeffrey S Gerber, Katie Chiotos

**Affiliations:** Baylor Scott and White Medical Center, Round Rock, Texas; University of Pennsylvania, Philadelphia, Pennsylvania; Children's Hospital of Philadelphia, Philadelphia, PA; Children's Hospital of Philadelphia, Philadelphia, PA

## Abstract

**Background:**

National guidelines recommend narrow-spectrum antibiotics for previously healthy children hospitalized with community-acquired pneumonia (CAP), but recommendations for medically complex children are not available.

**Figure:**
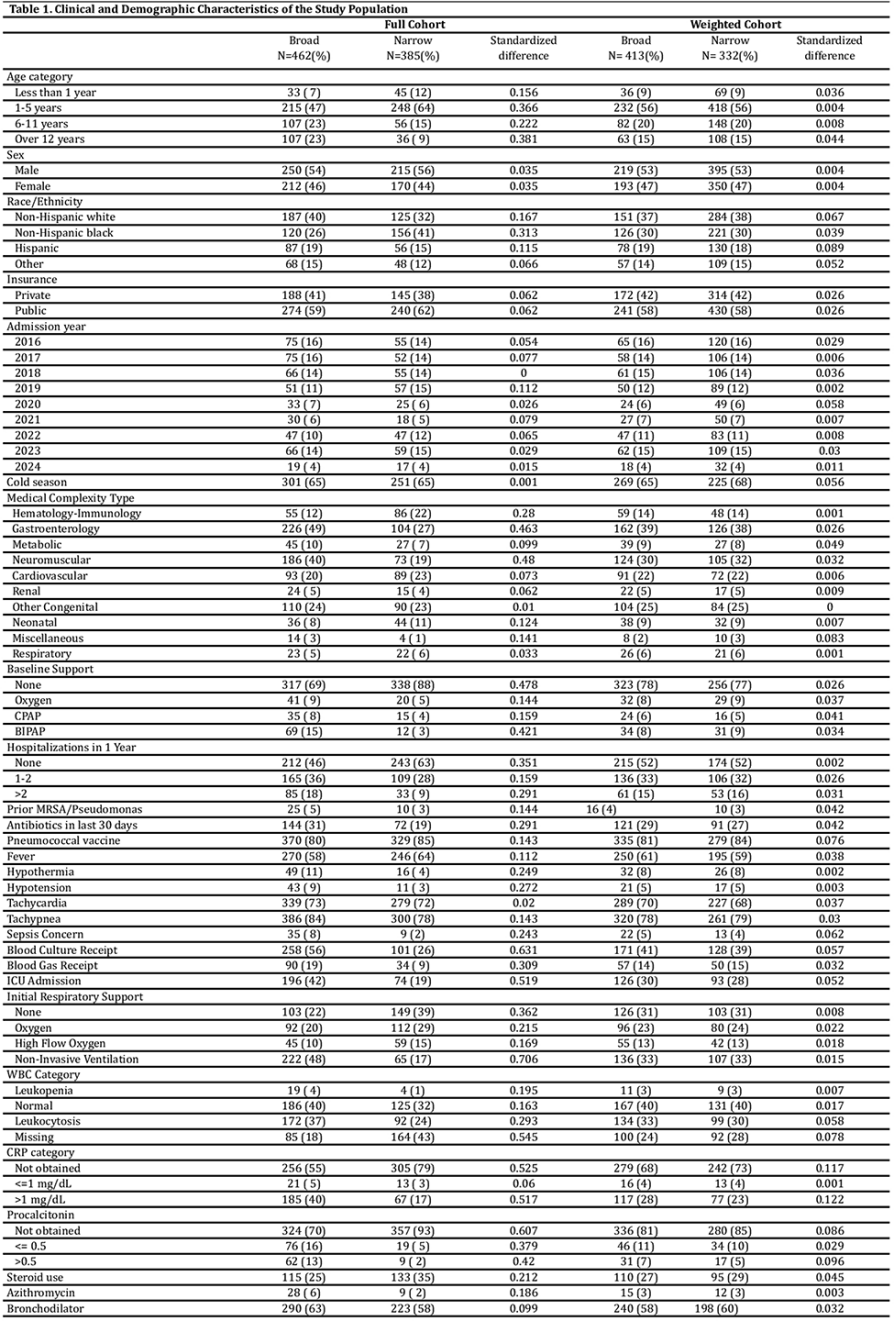

**Figure:**
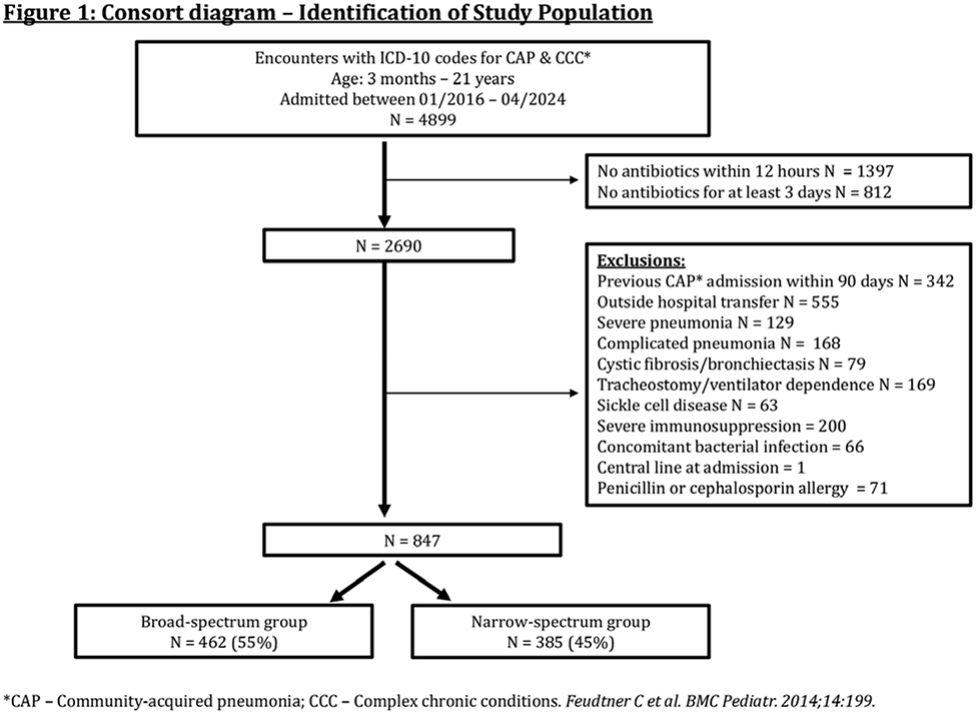

**Methods:**

We performed a retrospective cohort study comparing initial narrow- versus broad-spectrum antibiotics in medically complex children aged 3 months – 21 years admitted to an academic pediatric health system between 1/2016-4/2024. Children were included if they had an ICD-10 code for CAP, received at least 3 days of antibiotics, and had a complex chronic condition. We excluded children with severe or complicated CAP, cystic fibrosis, sickle cell disease, tracheostomy, severe immunosuppression, other bacterial infection, or a prior CAP admission within 90 days. The primary exposure was initial broad-spectrum antibiotics, defined as administration of any antibiotic except ampicillin or amoxicillin within 12 hours of presentation. The primary outcome was the time to discharge, censored at day 10. Secondary outcomes were severe pneumonia (new ICU transfer, intubation, or vasopressor use) and 30-day CAP-related revisits. Propensity scores were generated using generalized linear mixed models, and inverse probability of treatment weighting was used to mitigate confounding. Cumulative incidence curves and a flexible Royston-Palmer survival model were used to analyze time to discharge. Weighted logistic regression was used for binary outcomes.

**Figure:**
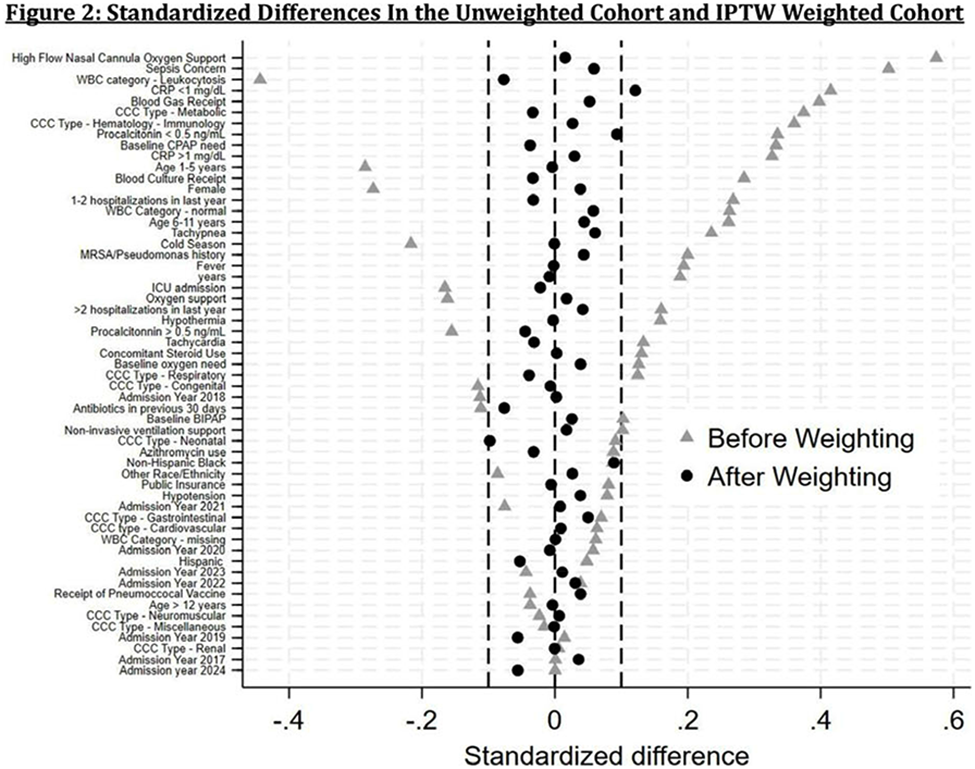

**Figure:**
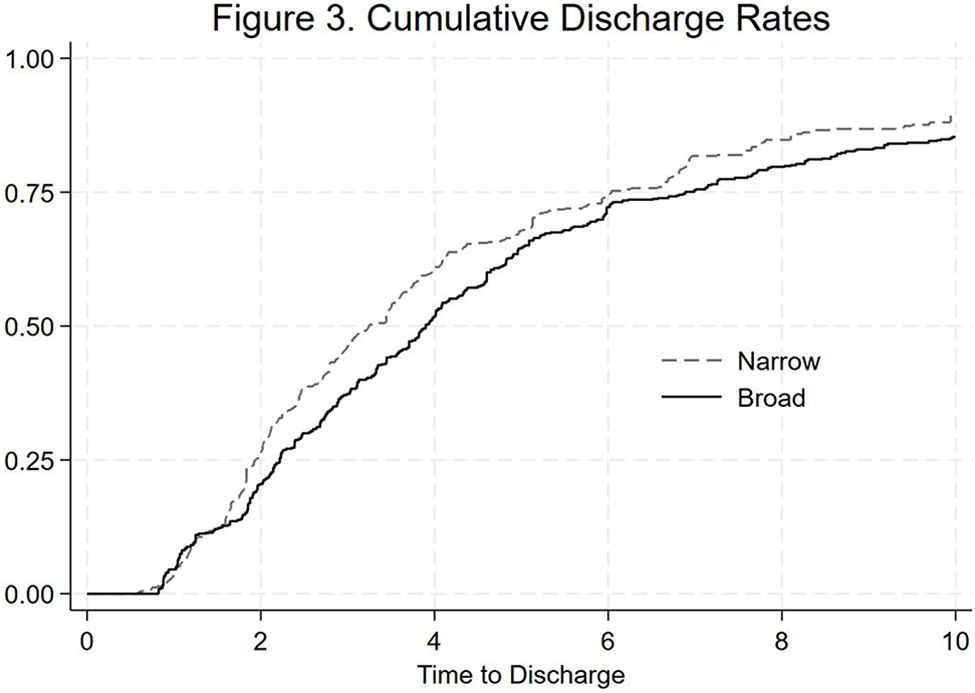

**Results:**

A total of 847 encounters (749 patients) met the inclusion criteria (Figure 1). After weighting and trimming to the area of common support, 754 encounters remained with adequate covariate balance (Table 1, Figure 2). The probability of being discharged by day 10 was 85% in the broad- and 90% in the narrow-spectrum group (Figure 3). There was no significant difference in time to discharge (hazard ratio, 0.84; 95% CI, 0.67–1.05), progression to severe pneumonia (OR, 1.29; 95% CI, 0.7-2.4), or 30-day CAP-related revisits (OR, 0.39; 95% CI, 0.1–1.2).

**Conclusion:**

In medically complex children hospitalized with non-severe CAP, no significant difference in outcomes was observed comparing initial narrow- and broad-spectrum antibiotics, supporting the use of initial narrow-spectrum antibiotics.

**Disclosures:**

All Authors: No reported disclosures

